# The Association Between Solid Fuel Use and Visual Impairment Among Middle-Aged and Older Chinese Adults: Nationwide Population-Based Cohort Study

**DOI:** 10.2196/43914

**Published:** 2023-07-26

**Authors:** Qingqing Jiang, Shiqi Wang, Hao Zhang, Yan Guo, Yiling Lou, Shen Huang, Qiqi You, Shiyi Cao

**Affiliations:** 1 School of Public Health Tongji Medical College Huazhong University of Science and Technology Wuhan China

**Keywords:** indoor air pollution, solid fuel, visual impairment, cohort study, Chinese

## Abstract

**Background:**

Indoor air pollution has been reported to have adverse effects on the eye; however, the health effects of exposure to cooking with solid fuels on visual impairment remain unclear in China.

**Objective:**

We aimed to examine the association between cooking with solid fuels and visual impairment, including distance visual impairment (DVI) and near visual impairment (NVI).

**Methods:**

Data were obtained from the China Health and Retirement Longitudinal Study, a nationwide survey of adults aged over 45 years who were enrolled in 2011 (Wave 1) and followed up in Wave 2 (2013), Wave 3 (2015), and Wave 4 (2018). We used Cox proportional hazards models to determine the association between solid fuels use and visual impairment. Additionally, the impact of switching cooking fuel types on vision function were examined through wave-specific data analysis (Wave 1 and Wave 4). Interaction and subgroup analyses were performed to explore the potential effect modifiers. Data were collected using the stratified multistage random sampling method and further analyzed using SPSS 27.0 and R 4.2.1 statistical software packages.

**Results:**

A total of 9559 middle-aged and older Chinese adults without visual impairment at baseline were included in the study, with 51.2% (n=4914) of the participants reporting that they cooked with solid fuels. During the follow-up period, 2644 (27.5%) and 3130 (32.6%) participants developed DVI and NVI, respectively. Compared with the clean fuel users, participants who cooked with solid fuels had a higher risk of DVI (hazards ratio [HR] 1.38, 95% CI 1.28-1.50) and NVI (HR 1.18, 95% CI 1.10-1.27). In addition, switching the cooking fuel type from clean to solid fuels was associated with an elevated risk of DVI (HR 1.51, 95% CI 1.15-1.98) and NVI (HR 1.39, 95% CI 1.06-1.82) compared to persistently using clean fuels during the follow-up period, although no protective effect of switching from solid to clean fuels on NVI was found (*P*=.52). In subgroup analysis, we found that cooking with solid fuels increased the risk of DVI in participants younger than 65 years (HR 1.41, 95% CI 1.28-1.55), men (HR 1.45, 95% CI 1.28-1.65), urban residents (HR 1.41, 95% CI 1.08-1.75), and smokers (HR 1.43, 95% CI 1.25-1.64). By contrast, negative effects of cooking with solid fuels on NVI were found in nonsmokers (HR 1.21, 95% CI 1.11-1.33) and urban residents (HR 1.20, 95% CI 1.10-1.37).

**Conclusions:**

Cooking with solid fuels was associated with an increased risk of visual impairment among middle-aged and older Chinese adults. These findings indicate that promoting the utilization of clean fuels is conducive to reducing the burden of visual impairment for the public.

## Introduction

Incomplete and inefficient combustion of solid fuels (including biomass and coal) is the primary cause of indoor air pollution, affecting approximately half the global population [1]. Prolonged exposure to indoor air pollution has many negative impacts on individuals’ health, such as causing respiratory disease, cancer, and eye problems, and is the foremost cause of premature death in developing countries [2]. Among the many human organs that could be affected by indoor air pollution, the eyes are directly exposed to the emissions from solid fuel combustion, including high levels of fine particulate matter (PM_2.5_) and carbon monoxide. These emissions would stimulate the production of reactive oxygen species, which can seriously impair the viability of eye cells and accelerate oxidation of the lens [3]. Moreover, the hazardous materials resulting from solid fuel combustion can also affect the body’s cardiovascular circulation system, leading to increased intraocular pressure through the aqueous humor.

Eye problems such as tearing, redness, itching, and stinging are widely reported to be linked to indoor air pollution [3,4]. A recent large cohort study provided evidence that prolonged exposure to indoor air pollution, which was mainly attributed to cooking with solid fuels, would increase the risk of major eye diseases (eg, conjunctiva disorders, cataracts) [5]. Moreover, long-term exposure to eye problems and eye diseases could result in severe visual impairment and even blindness [6-8]. Based on these findings [3-8], it is hypothesized that indoor air pollution exposure may be an environmental risk factor associated with visual impairment.

An estimated 2.2 billion people globally have visual impairments, with nearly half of these cases being preventable or untreated [9]. Untreated vision loss could have significant and lasting effects on a person’s ability to perform daily activities and access public services [10]. In China, the most populous country in the word, 30%-50% of adults over the age of 80 years and 7%-20% of adults over the age of 50 years were estimated to have visual impairments [11], ranking second in years lived with disability among all health impairments. As the population is aging rapidly, the burden of visual impairment is predicted to continue to rise [10,12].

Given the widespread use of solid fuels in China [13], evidence on the association of solid fuel use and visual impairment is urgently needed for policy makers to take effective actions to relieve the burden of visual impairment by promoting cleaner household energy.

To fill the above knowledge gap on the link between solid fuel use and visual impairment among Chinese adults, we performed this study to assess the longitudinal association between self-reported cooking with solid fuels and visual impairment in a nationally representative sample obtained from the China Health and Retirement Longitudinal Study (CHARLS). This prospective cohort study assessed the impact of indoor air pollution on visual impairment among middle-aged and older Chinese adults from 2011 to 2018 for the first time and also examined the effects of switching cooking fuel types on vision function over time.

## Methods

### Study Population

This nationwide population-based cohort study utilized data from the CHARLS, which was implemented by the National School of Development of Peking University. An unbiased and representative sample was obtained by proportional and multistage stratified sampling [14]. In the first stage, all counties in China were stratified by region. Regions were divided by urban districts or rural counties and per capita statistics on gross domestic product. Using the probability-proportional-to-size sampling technique, 150 county-level units were randomly chosen from the sampling frame. In the second stage, three primary sampling units (PSUs) were selected in each county based on a probability proportional to the population size. In the third stage, a random sample of 24 households was selected among all the households mapped in each selected PSU. Finally, for each selected household, one resident aged ≥45 years and their spouse were randomly selected as the participants in the survey. Briefly, the CHARLS survey included Chinese residents from 450 communities covering 150 county-level units in 28 provinces. From May 2011 to March 2012, a total of 17,708 residents with a response rate above 80% participated in the baseline survey (Wave 1). CHARLS respondents are followed every 2 years through a face-to-face computer-assisted personal interview to obtain individual information on sociodemographic characteristics, lifestyle behaviors, indoor air pollution, health status, and other factors of interest. Interviewers helped respondents who had sight problems by reading the questions in the standardized questionnaire during the interview process. To date, follow-up survey data are available for Wave 2 (2013), Wave 3 (2015-2016), and Wave 4 (2018) of CHARLS.

**Figure 1:**
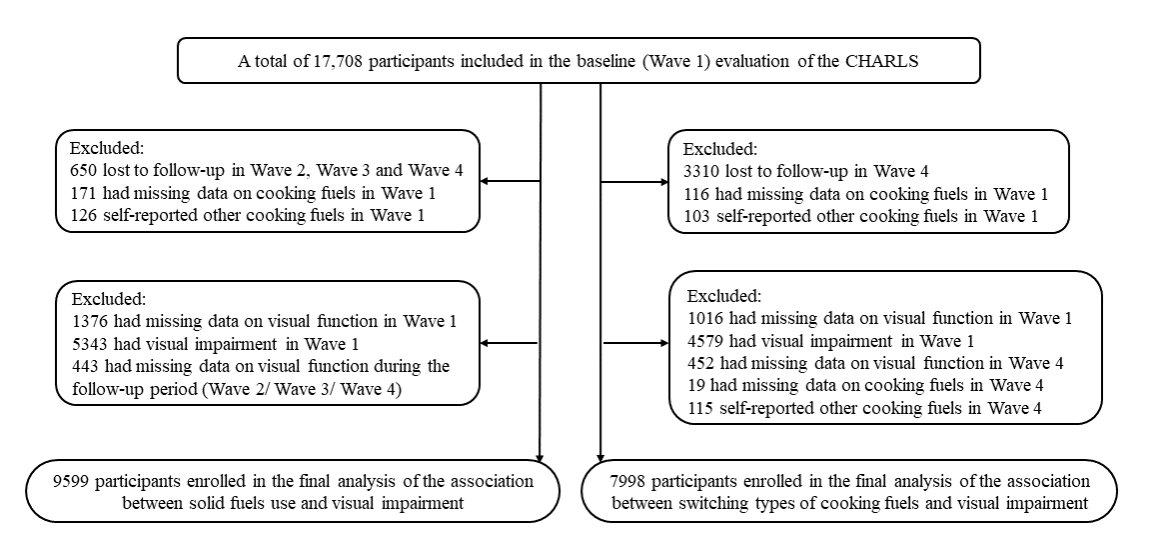
Flowchart of the selection process of participants. CHARLS: China Health and Retirement Longitudinal Study.

We used the data from all four waves to examine the relationship between solid fuel use and visual impairment; a total of 9559 individuals were selected based on the following exclusion criteria: (1) 650 lost to follow-up in Wave 2, Wave 3, and Wave 4; (2) 171 had missing data on cooking fuels in Wave 1; (3) 126 self-reported other cooking fuels in Wave 1; (4) 1376 had missing data on visual function in Wave 1; (5) 5343 had visual impairment in Wave 1; and (6) 443 had missing data on visual function during the follow-up period (Wave 2/Wave 3/Wave 4) (Figure 1).

In addition, we used the wave-specific data of Wave 1 and Wave 4 to examine the association between switching the fuel type and visual impairment; 7998 individuals were selected based on the following exclusion criteria: (1) 3310 lost to follow-up in Wave 4; (2) 116 had missing data on cooking fuels in Wave 1; (3) 103 self-reported other cooking fuels in Wave 1; (4) 1016 had missing data on visual function in Wave 1; (5) 4579 had visual impairment in Wave 1; (6) 452 had missing data on visual function in Wave 4; (7) 19 had missing data on cooking fuels in Wave 4; and (8) 115 self-reported other cooking fuels in Wave 4.

### Vision Assessment

Individuals’ distance vision was assessed by self-reported visual status when recognizing friends across the street (while wearing corrective lenses or glasses, if applicable) and personal near vision was assessed by self-reported visual status when reading regular newspapers (while wearing glasses or corrective lenses, if applicable). Possible responses to these questions were “poor,” “fair,” “good,” “very good,”, and “excellent.” Those reporting “poor” for near and distance vision were defined as having near visual impairment (NVI) and distance visual impairment (DVI), respectively. Those reporting “excellent,” “very good,” “good,” or “fair” were defined as having no visual impairment (reference group). Visual impairment assessment and categorization were performed in accordance with previous studies [15-17].

### Assessment of Cooking With Solid Fuels

The question “What are your main sources of cooking fuels?” was used to assess indoor cooking fuel types. At baseline, reporting “coal,” “crop residue,” and “wood” was defined as cooking with solid fuels. The main types of solid fuels were classified into coal and biomass (“crop residue” and “wood”). Reporting “liquefied petroleum,” “natural gas,” “electricity,” and “marsh gas” was defined as cooking with clean fuels. We compared data from respondents who reported cooking with clean fuels to those of the solid fuel users. In addition, we collected information on self-reported cooking fuel types in Wave 4, which had an additional choice of “do not cook” compared to the answers regarding the baseline question. We categorized “do not cook” as clean fuel, and assessed the association of switching cooking fuel types with visual impairment. Therefore, participants could be classified as switching from solid fuels to clean fuels or from clean fuels to solid fuels.

### Covariates

Baseline demographics (including age, gender, marital status, education level, residence) and health-related status (including smoking status, alcohol drinking, sleep duration, BMI, and chronic diseases) were included as covariates in our analyses. We classified cohabitation and marriage as married. Education level was divided into illiteracy (0 years) and others (≥1 year). Residence was divided into rural and urban areas [18]. BMI was calculated as weight divided by the square of height (kg/m^2^) [19]. Alcohol drinking was classified into drinker and nondrinker. Smoking status was classified into smoker and nonsmoker. Dyslipidemia, chronic lung disease, hypertension, heart disease, diabetes, and cancer were identified by self-reported physician diagnoses.

### Statistical Analysis

The baseline characteristics are described according the type of cooking fuels. Continuous variables were summarized as mean (SD) and were compared with independent *t* tests. Categorical variables were summarized as frequencies (percentages) and were compared with the *χ*^2^ test. A Cox proportional hazard model was performed to assess the effects of solid fuel use on visual impairment. We also compared data of participants who switched from clean fuels to solid fuels or from solid fuels to clean fuels with those who persistently used clean or solid fuels through wave-specific analysis. The proportional hazard assumption was examined using the Schoenfeld residual test. Model 1 was an unadjusted model. Model 2 was adjusted for age, gender, BMI, marital status, education level, residence, alcohol drinking, smoking status, and sleep duration. Model 3 was further adjusted for hypertension, dyslipidemia, diabetes, cancer, chronic lung disease, and heart disease based on Model 2. The percentage of missing covariates was less than 5% for all covariates, which conformed to an arbitrary missingness pattern. We used multiple imputation to account for these missing values. The Markov chain Monte Carlo method was used to analyze the complete data set and interpolate each missing value five times to obtain five complete data sets. Statistical analysis was then performed on each filled data set separately and the combined results of the five analyses were used to select the data set with the largest relative efficiency value. Additionally, we performed subgroup analyses and interactions analysis stratified by age, sex, residence area, and smoking status to assess potential effect modifications. The sensitivity analysis was conducted by excluding participants who reported not cooking in Wave 4 to ensure that our findings were robust. Cox proportional hazards model results are presented as hazard ratios with 95% CIs. Statistical analyses were performed using SPSS 27.0 and R 4.2.1 software. Two-tailed *P* values less than .05 were considered statistically significant.

### Ethical Considerations

The CHARLS was approved by the Research Ethics Committee of Peking University (IRB00001052-11015). This survey was anonymous and the answers are protected by privacy law. Written informed consent clarifying the study purposes was obtained from each participant before completing the interview.

## Results

### Characteristics of the Study Participants

A total of 9559 participants without visual impairment at baseline were included. The average age of included participants was 57.7 (SD 9.5) years and 50.3% of them were women. Overall, 51.2% (n=4914) of the participants primarily cooked with solid fuels (Table 1), and 15.3% (n=1469), 13.6% (n=1309), 18.4% (n=1770), 1.4% (n=137), 10.8% (n=1035), and 40.5% (n=3879) of the participants reported cooking with liquefied petroleum, natural gas, electricity, marsh gas, coal, and crop residue/wood burning at baseline, respectively. However, only 32.1% of the participants reported cooking with solid fuels in Wave 4, including 5.6% cooking with coal and 26.5% cooking with crop residue/wood burning (see Multimedia Appendix 1). In general, participants who reported cooking with solid fuels at baseline were more likely to be current smokers and reported less sleep time, lower educational levels, and lower BMI compared with clean fuel users. Significantly more participants living in rural areas and those suffering from chronic lung diseases used solid fuels (both *P*<.001). Additionally, significantly more individuals with hypertension, diabetes, and dyslipidemia used clean fuels for cooking rather than solid fuels (*P*=.04, *P*<.001, and *P*<.001, respectively).

**Table 1:** Baseline characteristics of the participants according to cooking fuel type.

Characteristic			Total (N=9599)		Solid fuels (n=4914)		Clean fuels (n=4685)		*P* value for difference	
Age (years), mean (SD)			57.7 (9.5)		58.3 (9.6)		56.9 (9.5)		.18	
**Sex, n (%)**										.57
	Male	4768 (49.7)		2455 (50.0)		2313 (49.4)				
	Female	4831 (50.3)		2459 (50.0)		2372 (50.6)				
**Education (years), n (%)**										<.001
	0	2250 (23.4)		1508 (30.7)		742 (15.8)				
	≥1	7349 (76.6)		3406 (69.3)		3943 (84.2)				
**Marital status, n (%)**										.37
	Married	8596 (89.6)		4387 (89.3)		4209 (89.8)				
	Others	1003 (10.4)		527 (10.7)		476 (10.2)				
**Residence, n (%)**										<.001
	Rural	7186 (74.9)		4570 (93.0)		2616 (55.8)				
	Urban	2413 (25.1)		344 (7.0)		2069 (44.2)				
BMI (kg/m^2^), mean (SD)^a^			23.4 (3.3)		23.1 (3.4)		23.7 (3.2)		<.001	
**Smoking status, n (%)**										<.001
	Smoker	3794 (39.5)		2038 (41.5)		1756 (37.5)				
	Nonsmoker	5805 (60.5)		2876 (58.5)		2929 (62.5)				
**Alcohol consumption, n (%)**										.18
	Drinker	3297 (34.3)		1656 (33.7)		1641 (35.0)				
	Nondrinker	6302 (65.7)		3258 (66.3)		3044 (65.0)				
**Sleep duration (hours), n (%)^b^**										.001
	<7	4470 (46.9)		2367 (48.5)		2103 (45.2)				
	≥7	5068 (53.1)		2515 (51.5)		2553 (54.8)				
**Hypertension, n (%)^c^**										.04
	Yes	2099 (22.0)		1030 (21.1)		1069 (22.9)				
	No	7453 (78.0)		3851 (78.9)		3602 (77.1)				
**Diabetes, n (%)^d^**										<.001
	Yes	439 (4.6)		174 (3.6)		265 (5.7)				
	No	9077 (95.4)		4689 (96.4)		4388 (94.3)				
**Dyslipidemia, n (%)^e^**										<.001
	Yes	826 (8.7)		327 (6.8)		499 (10.8)				
	No	8616 (91.3)		4488 (93.2)		4128 (89.2)				
**Cancer, n (%)^f^**										.50
	Yes	79 (0.8)		37 (0.8)		42 (0.9)				
	No	9480 (99.2)		4849 (99.2)		4631 (99.1)				
**Chronic lung disease, n (%)^g^**										<.001
	Yes	844 (8.8)		512 (10.5)		332 (7.1)				
	No	8718 (91.2)		4378 (89.5)		4340 (92.9)				
**Heart disease, n (%)^h^**										.23
	Yes	986 (10.3)		486 (10.0)		500 (10.7)				
	No	8564 (89.7)		4398 (90.0)		4166 (89.3)				

^a^Data missing from 425 participants.

^b^Data missing from 61 participants.

^c^Data missing from 47 participants.

^d^Data missing from 83 participants.

^e^Data missing from 157 participants.

^f^Data missing from 40 participants.

^g^Data missing from 37 participants.

^h^Data missing from 49 participants.

### Association Between Cooking With Solid Fuels and Visual Impairment

During the follow-up period, 2644 (27.5%) and 3130 (32.6%) of the 9599 total participants reported DVI and NVI, respectively (Table 2). The effects of cooking with solid fuels on visual impairment are presented in Table 2. In the crude model, using solid fuels for cooking elevated the risk of DVI and NVI. The findings from Model 1 and Model 2 were consistent with those of the crude model. After adjusting for extensive confounders (Model 3), we found that participants who cooked with solid fuels had a significantly increased risk of DVI and NVI. In addition, compared with the clean fuel users, the participants who cooked with coal had a significantly increased risk of DVI and NVI. Moreover, cooking with biomass significantly increased the risk of DVI and NVI (see Multimedia Appendix 2).

Through the wave-specific data, we found that participants who switched their cooking type from clean to solid fuels had a 51% elevated risk of DVI and a 39% elevated risk of NVI compared with the persistent clean fuel users. However, there was no evidence of an impact of switching from solid to clean fuels on NVI (*P*=.52). After excluding participants who reported not cooking in Wave 4, our findings remained robust (see Multimedia Appendix 3).

**Table 2:** Longitudinal association of cooking fuel type, switching fuel type, and visual impairment among middle-aged and older Chinese adults.

Variable					Participants, n		Events/incidence rate (per 1000 person-years)		Model 1^a^, HR^b^ (95% CI)		Model 2^c^, HR (95% CI)			Model 3^d^, HR (95% CI)	
**Distance visual impairment (DVI)**															
	**Cooking fuel type (N=9559)**														
		Clean fuel (reference)		4685		1064 (39.8)		N/A^e^		N/A			N/A		
		Solid fuel		4914		1580 (58.1)		1.42 (1.31-1.53)		1.36 (1.26-1.47)			1.38 (1.28-1.50)		
	**Switching fuel type (N=7998)**														
		**From solid to clean fuels**													
			Persistent use of solid fuels (reference)	2237		462 (29.6)		N/A		N/A			N/A		
			Solid to clean fuels use	1931		338 (25.0)		0.84 (0.73-0.96)		0.85 (0.74-0.98)			0.85 (0.74-0.98)		
		**From clean to solid fuels**													
			Persistent use of clean fuels (reference)	3496		419 (17.2)		N/A		N/A			N/A		
			Clean to solid fuels use	334		62 (26.6)		1.60 (1.22-2.09)		1.48 (1.13-1.93)			1.51 (1.15-1.98)		
**Near visual impairment (NVI)**															
	**Cooking fuel type (N=9559)**														
		Clean fuel (reference)		4685		1371 (53.4)		N/A		N/A			N/A		
		Solid fuel		4914		1759 (65.8)		1.20 (1.11-1.28)		1.18 (1.10-1.26)			1.18 (1.10-1.27)		
	**Switching fuel type (N=7998)**														
		**From solid to clean fuels**													
			Persistent use of solid fuels (reference)	2237		414 (26.5)		N/A		N/A			N/A		
			Solid to clean fuels use	1931		346 (25.6)		0.96 (0.83-1.10)		0.96 (0.83-1.10)			0.96 (0.83-1.11)		
		**From clean to solid fuels**													
			Persistent use of clean fuels (reference)	3496		456 (18.7)		N/A		N/A			N/A		
			Clean to solid fuels use	334		61 (26.2)		1.44 (1.10-1.88)		1.38 (1.06-1.81)			1.39 (1.06-1.82)		

^a^Model 1: Unadjusted.

^b^HR: hazard ratio.

^c^Model 2: Adjusted for age, gender, BMI, marital status, years of education, residence, smoking status, alcohol consumption, and sleep duration.

^d^Model 3: Further adjusted for hypertension, dyslipidemia, diabetes, cancer, chronic lung disease, and heart disease based on Model 2.

^e^N/A: not applicable.

### Subgroup Analyses on the Association Between Cooking With Solid Fuels and Visual Impairment

We performed analyses of interactions and subgroups on the relationship between using solid fuels for cooking and visual impairment in Model 3 (Table 3). We found that cooking with solid fuels had an increased risk of DVI among participants younger than 65 years, men, urban residents, and smokers. However, cooking with solid fuels resulted in a higher risk of NVI for nonsmokers and urban residents.

**Table 3:** Subgroup analysis of the association between solid fuel use and visual impairment among middle-aged and older Chinese adults.

Subgroups		Cooking with solid fuel, n	DVI^a^							NVI^b^				
			Events, n		HR^c^ (95% CI)^d^		*P* value for interaction		Events, n		HR (95% CI)^d^		*P* value for interaction	
**Age (years)**								<.001						.06
	<65	3657	1082		1.41 (1.28-1.55)				1313		1.17 (1.08-1.26)			
	≥65	1257	498		1.27 (1.10-1.48)				446		1.21 (1.04-1.42)			
**Sex**								.01						.17
	Male	2455	667		1.45 (1.28-1.65)				806		1.19 (1.07-1.33)			
	Female	2459	913		1.34 (1.21-1.48)				953		1.16 (1.06-1.28)			
**Residence**								<.001						<.001
	Urban	344	83		1.41 (1.08-1.75)				93		1.20 (1.10-1.37)			
	Rural	4570	1497		1.16 (1.06-1.27)				1666		1.05 (0.97-1.14)			
**Smoking status**								<.001						<.001
	Smoker	2038	585		1.43 (1.25-1.64)				678		1.12 (0.99-1.26)			
	Nonsmoker	2876	995		1.36 (1.23-1.49)				1081		1.21 (1.11-1.33)			

^a^DVI: distance visual impairment.

^b^NVI: near visual impairment.

^c^HR: hazard ratio.

^d^Adjusted for age, gender, BMI, marital status, years of education, residence, smoking status, alcohol consumption, sleep duration, hypertension, dyslipidemia, diabetes, cancer, chronic lung disease, and heart disease.

## Discussion

### Summary of Findings

This study demonstrated that cooking with solid fuels increased the risks of visual impairment, with an adverse effect of solid fuel use found for both DVI and NVI, especially for participants living in urban areas. Moreover, switching from clean to solid fuels was demonstrated to increase the risk of DVI and NVI compared to the persistent use of clean fuel. These main findings are consistent with evidence from previous studies conducted in countries neighboring China. In India, the older population, who are highly reliant on using unclean cooking fuel, had a higher prevalence of visual impairment [20]. In Nepal, the incidence of cataracts was two-fold higher in women using biomass for cooking compared to that of women using clean fuel [21]. This study thus adds to the accumulating evidence of adverse vision health consequences potentially arising from the use of unclean cooking fuels in low- and middle-income countries [22].

The essential mechanisms of the impacts of solid fuel utilization on visual impairment are undefined [23]. One possible reason is that burning solid fuels produces high levels of gaseous pollutants that increase the risk of eye diseases and may lead to vision impairment. On the one hand, PM_2.5_ is the primary pollutant in solid fuel smog, which represents a mixture of thousands of harmful chemicals such as heavy metals and polycyclic aromatic hydrocarbons [24]. A previous study showed that for each 1 μg/m^3^ rise in ambient PM_2.5_ exposure, the risk of glaucoma increased by 6% [25]. Free radicals accelerate the oxidation of the lens when the eye organ is directly exposed to the fumes of solid fuel, which results in an increased risk of cataracts [26] as the leading cause of vision impairment or loss worldwide [27,28]. On the other hand, incomplete combustion of solid fuel produces carbon monoxide, which is harmful to the eyes due to hypoxia [29]. Another possibility is that indoor air pollutants resulting from solid fuel combustion can affect the body’s cardiovascular system and the effect on the aqueous humor can lead to increased intraocular pressure. Moreover, when burning solid fuels, the anterior eyes are more susceptible to damage from sparks, wood dust, or sharp wood and the consequent ocular trauma would increase the risk of vision impairment [30,31]. Heat exposure associated with burning solid fuels may be another potential risk factor for eye diseases [32,33], which would contribute to the development of age-related cataracts and the early onset of presbyopia if the transient temperature elevation in the lens recurs over many years. In addition, previous studies have linked long-term solid fuel use to diabetes [34], depression [35], and cognitive impairment [36], which were reported as potential risk factors affecting vision health.

Previous studies observed that women were more likely to develop eye diseases linked to solid fuel use [37-39], suggesting that as women traditionally played a vital role in cooking, they had observably higher indoor air pollution exposure. However, our study showed that men cooking with solid fuels appeared to be at a higher risk of DVI than women. This result is consistent with the cross-sectional study from India, which failed to find that the visual impairment risk associated with use of unclean cooking fuels was higher for women and in households without a separate kitchen or ventilation [20]. One possible reason for this inconsistency might be differences in the study populations. In our study, most of the individuals were retirees aged over 60 years, who tend to spend most of their time at home, which would increase the frequency of couples cooking together. This situation thereby increases the exposure to indoor air pollution caused by the use of solid fuels for men. Moreover, there is no doubt that the smoking rate is much higher among men than among women and there may be an interaction between vision impairment and solid fuel use due to the apparent gender difference in smoking habits.

More harmful impacts of cooking with solid fuels on NVI were noted among urban residents. Rural areas are usually less economically developed and have a lower population density than urban areas. Higher industrialization in urban areas may increase the risk of visual impairment since rapid industrialization would result in worse indoor air pollution and associated health outcomes [40]. Additionally, the higher population density in urban areas leads to a lower living space per capita, which may elevate the indoor pollutant concentration [41]. Despite some households having installed ventilation devices to prevent severe indoor air pollution, the level of air pollutants in domestic kitchens using solid fuels remains high [42]. In addition, switching cooking fuels from solid to clean fuels failed to decrease the risks of cataracts and conjunctiva disorders compared to the persistent use of solid fuels [5]. Taking this into consideration, it is necessary to call on the general public to use clean energy to reduce indoor air pollution and promote healthy vision, especially in rural areas.

Simultaneously, an interesting result emerging from our study was that switching from clean fuels to solid fuels could increase the risk of visual impairment compared with the continued use of clean fuels. The switch of cooking fuels from clean fuels to biomass fuels might be related to the migration from urban to rural areas among older people in China, which has been a prevalent trend in recent years [43,44]. Older adults generally prefer solid fuels more than younger adults and they are consequently more prone to the adverse effects of indoor air pollution. A cohort study with a 3-year follow-up period found that cooking with biomass fuels was associated with a higher risk of visual impairment among older Chinese adults with a mean baseline age of 82.56 years [43]. The persistent formation of lens fibers and the relative thickening of the lens cortex are important features during the aging process, resulting in changes in the refractive index of the lens [45]. This would in turn result in a higher risk of visual impairment when older adults change their cooking fuels from clean to solid fuels as their eyes may not be able to adapt to the sudden deterioration of air quality caused by solid fuels. Therefore, public health policies and investments that support cleaner household energy can effectively reduce the key sources of ambient air pollution, especially by avoiding the persistent use of solid fuels or switching from clean fuels to solid fuels, to further relieve the burden on visual impairment.

### Limitations

This study has several limitations. First, with the rapid development of modernization, urbanization, and industrialization in China over the past three decades, more and more rural residents have gradually begun to use clean energy. Some of the participants in this study may have switched from solid to clean fuels prior to the baseline assessment, which would lead to an underestimate of the impact of solid fuels on visual function. Second, individual visual acuity and the exposure level to household pollutants cannot be accurately estimated due to data unavailability; thus, further research should use an objective assessment to verify the data and adjust for lead-time bias [46]. Third, there remain some potential confounders that we were unable to measure that could also have an impact, including occupational dust [31], heat exposure [33], or sunlight [47].

### Conclusion

Overall, our study demonstrated that using solid fuels for cooking was significantly associated with elevated risks of visual impairment among Chinese adults aged over 45 years, especially among participants living in urban areas. Furthermore, switching the cooking fuel type from clean to solid fuels may also significantly increase the risk of visual impairment. Therefore, this study highlights the value of promoting the availability of household clean fuels and encouraging the consistent use of clean fuels to decrease the burden of visual impairment.

## Appendix

Multimedia Appendix 1 The frequency of cooking fuel types of the study participants.

Multimedia Appendix 2 Longitudinal association between cooking fuel type and visual impairment among middle-aged and older Chinese adults.

Multimedia Appendix 3 The association of switching cooking fuels type with visual impairment excluding participants who reported not cooking in Wave 4.

## Abbreviations

CHARLS: China Health and Retirement Longitudinal Study
DVI: distance visual impairment
NVI: near visual impairment
PM2.5: fine particulate matter
PSU: primary sampling unit

## Data Availability

The original data set is publicly available at the China Health and Retirement Longitudinal Study (CHARLS) website [48]. The data analyzed in our study can be obtained from the corresponding author upon reasonable request.
