# Supplementary material for: The Association Between Solid Fuel Use and Visual Impairment Among Middle-Aged and Older Chinese Adults: Nationwide Population-Based Cohort Study
Source: JMIR Public Health Surveill. 2023 Jul 26;9:e43914. doi: 10.2196/43914 (PMC10413239; doi:10.2196/43914)
Supplement: Multimedia Appendix 1 [file publichealth_v9i1e43914_app1.docx]

**Multimedia Appendix 1.** The frequency of cooking fuel types of the study participants.

| ***Cooking fuels types*** |  | | **Frequency (N [%])** | |
| --- | --- | --- | --- | --- |
| ***Baseline* (N=9,559)** | | ***Source of cooking fuel*** | | |
| Clean fuel | 4685 (48.8) |  | |  |
|  |  | Liquefied petroleum | | 1469 (15.3) |
|  |  | Natural gas | | 1309 (13.6) |
|  |  | Electricity | | 1770 (18.4) |
|  |  | Marsh gas | | 137 (1.4) |
| Solid fuel | 4914 (51.2) |  | |  |
|  |  | Coal | | 1035 (10.8) |
|  |  | Crop residue/Wood burning | | 3879 (40.5) |
| ***Wave 4* (N=7,998)** | | ***Source of cooking fuel*** | | |
| Clean fuel | 5427 (67.9) |  | |  |
|  |  | Liquefied petroleum | | 1433 (17.9) |
|  |  | Natural as | | 2056 (25.7) |
|  |  | Electricity | | 1853 (23.2) |
|  |  | Marsh gas | | 48 (0.6) |
|  |  | Do not cook | | 37 (0.5) |
| Solid fuel | 2571 (32.1) |  | |  |
|  |  | Coal | | 446 (5.6) |
|  |  | Crop residue/Wood burning | | 2125 (26.5) |
