# Supplementary material for: The Association Between Solid Fuel Use and Visual Impairment Among Middle-Aged and Older Chinese Adults: Nationwide Population-Based Cohort Study
Source: JMIR Public Health Surveill. 2023 Jul 26;9:e43914. doi: 10.2196/43914 (PMC10413239; doi:10.2196/43914)
Supplement: Multimedia Appendix 2 [file publichealth_v9i1e43914_app2.docx]

**Multimedia Appendix 2. Longitudinal association between cooking fuel type and visual impairment among middle-aged and older Chinese.**

| **Cooking fuel type**  **(N=9,559)** | **N** | **Events/incidence rate** (per 1000 person-years) | **Model 1**  HR (95% CI) | **Model 2**  HR (95% CI) | **Model 3**  HR (95% CI) |
| --- | --- | --- | --- | --- | --- |
| **Distance visual impairment (DVI)** | | | | | |
| ***Fuel type*** | | | | | |
| Clean fuel | 4685 | 1064 (39.8) | Ref | Ref | Ref |
| Coal | 1035 | 319 (55.9) | 1.32 (1.17-1.50) *** | 1.27 (1.12-1.44) *** | 1.28 (1.13-1.46) *** |
| Biomass | 3879 | 1261 (58.8) | 1.44 (1.33-1.56) *** | 1.39 (1.28-1.51) *** | 1.41 (1.30-1.53) *** |
| **Near visual impairment (NVI)** | | | | | |
| ***Fuel type*** | | | | | |
| Clean fuel | 4685 | 1371 (53.4) | Ref | Ref | Ref |
| Coal | 1035 | 371(66.2) | 1.17 (1.04-1.31) ** | 1.16 (1.03-1.30) * | 1.16 (1.04-1.31) * |
| Biomass | 3879 | 1388 (65.7) | 1.20 (1.11-1.30) *** | 1.18 (1.10-1.28) *** | 1.18 (1.10-1.28) *** |

Abbreviations: CI, confidence interval; HR, hazard ratio; Ref., reference.

Model 1: Unadjusted.

Model 2: Adjusted for age, gender and body mass index, marital status, education year, residence, smoking status, alcohol consumption, sleep duration.

Model 3: Further adjusted hypertension, dyslipidemia, diabetes, cancer, chronic lung disease, heart disease based on Model 2.

**p* <0.05, ***p* <0.01, ****p* <0.001.
