# Supplementary material for: The Association Between Solid Fuel Use and Visual Impairment Among Middle-Aged and Older Chinese Adults: Nationwide Population-Based Cohort Study
Source: JMIR Public Health Surveill. 2023 Jul 26;9:e43914. doi: 10.2196/43914 (PMC10413239; doi:10.2196/43914)
Supplement: Multimedia Appendix 3 [file publichealth_v9i1e43914_app3.docx]

**Multimedia Appendix 3. The association of switching cooking fuels type with visual impairment excluding participants who reported not cooking.**

| **Cooking fuel type**  **(N=7,998)** | **Model 1**  HR (95% CI) | **Model 2**  HR (95% CI) | **Model 3**  HR (95% CI) |
| --- | --- | --- | --- |
| **Distance visual impairment (DVI)** | | | |
| ***Switching fuels type from solid to clean fuels*** | | | |
| Persistent use of solid fuels | Ref | Ref | Ref |
| Solid to clean fuels use | 0.84 (0.73-0.97) * | 0.85 (0.74-0.98) * | 0.83 (0.72-0.96) * |
| ***Switching fuels type from clean to solid fuels*** | | | |
| Persistent use of clean fuels | Ref | Ref | Ref |
| Clean to solid fuels use | 1.60 (1.22-2.08) *** | 1.47 (1.13-1.93) ** | 1.51 (1.15-1.98) ** |
| **Near visual impairment (NVI)** | | | |
| ***Switching fuels type from solid to clean fuels*** | | | |
| Persistent use of solid fuels | Ref | Ref | Ref |
| Solid to clean fuels use | 0.96 (0.83-1.11) | 0.95 (0.83-1.11) | 0.95 (0.83-1.10) |
| ***Switching fuels type from clean to solid fuels*** | | | |
| Persistent use of clean fuels | Ref | Ref | Ref |
| Clean to solid fuels use | 1.43 (1.10-1.87) ** | 1.38 (1.05-1.80) * | 1.38 (1.06-1.81) * |

Abbreviations: CI, confidence interval; HR, hazard ratio; Ref., reference.

Model 1: Unadjusted.

Model 2: Adjusted for age, gender and body mass index, marital status, education year, residence, smoking status, alcohol consumption, sleep duration.

Model 3: Further adjusted hypertension, dyslipidemia, diabetes, cancer, chronic lung disease, heart disease based on Model 2.

**p* <0.05, ***p* <0.01, ****p* <0.001.
